# Measurable Residual Disease Analysis by Flow Cytometry: Assay Validation and Characterization of 385 Consecutive Cases of Acute Myeloid Leukemia

**DOI:** 10.3390/cancers17071155

**Published:** 2025-03-29

**Authors:** Husam A. Jum’ah, Gregory E. Otteson, Michael M. Timm, Matthew J. Weybright, Min Shi, Pedro Horna, Dragan Jevremovic, Kaaren K. Reichard, Horatiu Olteanu

**Affiliations:** Division of Hematopathology, Department of Pathology and Laboratory Medicine, Mayo Clinic, Rochester, MN 55905, USA; jumah.husam@mayo.edu (H.A.J.);

**Keywords:** acute myeloid leukemia, AML, bone marrow, measurable residual disease, MRD, flow cytometry

## Abstract

**Background/Objectives**: Acute myeloid leukemia (AML) is a biologically heterogeneous malignancy with a variable prognosis. Despite many patients achieving complete remission, relapse remains common, underscoring the need for effective prognostic markers. Measurable residual disease (MRD) has emerged as a critical prognostic indicator, associated with higher relapse risk and shorter survival. This study reports on our initial experience of MRD detection by flow cytometry in 385 bone marrow samples from 126 AML patients. **Methods**: The flow cytometry MRD assay, validated according to stringent consensus recommendations, consists of a 3-tube, 10-color panel incorporating a broad spectrum of lineage differentiation markers. Analytical specificity, sensitivity, precision, and reproducibility were evaluated, demonstrating the assay’s robustness. **Results**: The results reveal distinct immunophenotypic aberrancies in all AML cases, with consistent identification of aberrant immunophenotypes in follow-up specimens. AML MRD was detected in 32 out of 126 patients (25%) and in 77 out of 385 analyses (20%), with a median aberrant blast percentage of 1.87% (range, 0.01–12). A change in immunophenotype was documented in 21% of the MRD-positive cases. MRD positivity detected in the first sample studied was associated with reduced overall survival (HR: 5.153; *p* < 0.0001). **Conclusions**: Our findings support the integration of flow cytometric MRD analysis into routine clinical practice to enhance risk stratification and treatment planning for AML patients, as currently recommended by professional guidelines.

## Simple Summary

Measurable residual disease (MRD) in acute myeloid leukemia (AML) is defined as the presence of leukemic blasts below the morphologic threshold of complete remission (5%). The presence of MRD can be assessed by sensitive ancillary techniques, such as multiparameter flow cytometry, quantitative polymerase chain reaction, and next-generation sequencing has therapeutic and prognostic value for patients with AML. The use of flow cytometry for MRD assessment is limited in the USA due to a lack of standardization and the requirement of high analytic expertise. In this study, we present the characteristics of a sensitive flow cytometry assay for AML MRD assessment, based on current consensus recommendations. We also summarize data from a group of 126 patients and 385 consecutive MRD analyses by using this assay.

## 1. Introduction

Acute myeloid leukemia (AML) is a biologically heterogeneous malignancy of immature myeloid precursors, with a highly variable, and generally unfavorable prognosis in adults [1,2,3]. While most patients under the age of 60 achieve complete remission (CR), characterized by the absence of morphologic features of AML (<5% bone marrow blasts), relapse remains common. This results in significantly lower cure rates (5-year overall survival of 40–50%), compared to CR rates of 60–85% following induction chemotherapy [2,4,5,6]. Acute promyelocytic leukemia is a notable exception, highlighting the importance of prognostic markers, such as recurrent genetic and molecular abnormalities, in refining prognosis and guiding therapy in these patients. Over the past two decades, considerable evidence has accumulated supporting minimal/measurable residual disease (MRD) as a prognostic indicator, associated with higher relapse risk and shortened survival [6,7,8,9,10,11,12,13,14,15,16,17,18,19]. Furthermore, this parameter has been incorporated as a refined response criterion to therapy in both the 2018 and 2021 ELN recommendations for the diagnosis and management of adults with AML [2].

From a widespread adoption and practical implementation perspective, MRD analysis in AML lags behind the methodologies and harmonization protocols developed for B lymphoblastic leukemia (B-ALL) [20]. Although several detection methods may be used for evaluating MRD status in patients with AML, flow cytometry is applicable to most instances (>90% of patients) [10]. This technical advantage is challenged by the requirement of fresh sample and the complexity of immunophenotypic analysis needed to identify very low levels of abnormal myeloid blasts (typically 1 in 1000 cells, as currently recommended by guidelines), while also distinguishing these aberrant populations from the multitude of maturing hematopoietic precursors present in regenerating bone marrow biopsies. Additionally, the lack of standardization for AML MRD flow cytometry protocols in the United States and the scarcity of academic or reference laboratories with the technical infrastructure and expertise to perform this analysis accurately and reproducibly, further compounds the problem. To address this gap, the European LeukemiaNet MRD Working Party has issued consensus recommendations for technical best practices, including MRD analysis by flow cytometric and molecular analysis, the selection of appropriate time points, uniform reporting criteria, and the clinical implications of MRD assessment [21,22,23].

In this study, we report our initial single-institution experience of MRD detection by flow cytometry in 385 consecutive samples from a cohort of 126 patients with AML, with extensive longitudinal assessment. These results were obtained using a flow cytometry MRD assay validated according to stringent consensus recommendations from the ELN MRD Working Party [23] and assay performance criteria listed in the current CLSI-H62 guidelines [24]. We utilized a judiciously designed 3-tube, 10-color panel incorporating lineage differentiation markers recommended by 2021 ELN MRD, and assessed assay accuracy using a separate flow cytometry assay with comparable performance. We evaluated analytical specificity, analytical and functional sensitivity, precision/reproducibility, linearity, sample stability, and reportable reference range, as recommended by CLSI-H62 [24] and similar to a previous validation of a highly sensitive flow cytometry assay for MRD detection in multiple myeloma [25]. Our findings show that each case exhibits discernible immunophenotypic blast aberrancies. Additionally, we demonstrate that our flow MRD assay can consistently and accurately identify these aberrant immunophenotypes in follow-up specimens, based on a combination of “leukemia-associated immunophenotype (LAIP)” and “difference from normal (DfN)” approaches, irrespective of temporal variations in myeloblast antigen expression. Our results are considered within the context of modern diagnostic and therapeutic approaches to patients with AML, including the significance of MRD detection as an outcome predictor.

## 2. Patients and Methods

### 2.1. Patients

Consecutive bone marrow biopsies (*n* = 385) from 126 patients with a documented diagnosis of acute myeloid leukemia, further subclassified per World Health Organization (WHO) 2022 criteria [1], were collected at Mayo Clinic in Rochester, MN, between August 2021 and October 2023. In our practice, when a patient undergoes a bone marrow biopsy procedure, we collect both a bone marrow aspirate and a bone marrow core biopsy, in addition to a peripheral blood smear and clot section. We collected clinical and laboratory data from the electronic medical record, including demographic information, immunohistochemistry, cytogenetics/fluorescence in situ hybridization (FISH), and molecular/NGS testing results. This study was approved by the Mayo Clinic Institutional Review Board.

### 2.2. Morphology and Flow Cytometry

All cases were centrally reviewed by two of the authors (HS and HO) in a blinded manner. Morphology assessment included peripheral blood, bone marrow aspirate, and touch imprint preparation slides, as well as bone marrow core biopsy and clot sections on all patients.

Multiparametric flow cytometry (MFC) immunophenotyping for MRD analysis was performed on “first-pull” bone aspirate samples as previously described [26]. Briefly, we used a lyse–wash–stain procedure, with staining performed in three 10-color tubes, using antibodies against 18 unique antigens. The specific antibody combinations and corresponding fluorochromes are detailed in Table 1. No cell viability marker is included in the antibody panel. For sample processing, we start with a cell count of 20 million and add 2 million cells per tube for staining, based on the post-processing cell count. Target event collection is 1.2 million events per tube, using BD FACSLyric flow cytometers (BD, Franklin Lakes, NJ, USA) equipped with FACSuite software (https://www.bdbiosciences.com/en-us/products/software/instrument-software/bd-facsuite-application?tab=Overview) for data acquisition. MRD values are calculated by dividing the number of abnormal cells in each of the three antibody tubes by the total number of leukocytes. The total number of leukocytes (the denominator) consists of the total analyzed leukocytes, excluding debris, non-viable cells, aggregates, red blood cells, and electronic noise. The AML MRD antibody panel was designed based on consensus recommendations from the ELN MRD Working Party. It has a robust 5-marker backbone (consisting of CD34, CD117, CD13, CD45, and HLA-DR) and is intended for identifying abnormal myeloid, erythroid, and lymphoid precursors in bone marrow samples collected in three different anticoagulants (EDTA, ACD, and heparin). The assay was validated to a lower limit of quantification of 0.01% (as further described below), which is one log more sensitive than the ELN AML MRD guidelines [23]. The lower limit of quantification (LOQ) is based on a minimum of 500,000 events analyzed in each tube and an abnormal cell immunophenotype that is consistently and conservatively detected in a cluster composed of at least 50 events. Consistent performance is ensured by collecting a targeted 1,200,000 events per tube, with the goal of achieving 1,000,000 non-aggregate events per tube (a total of 3,000,000). In addition to the redundant 5-antigen backbone, the AML MRD antibody panel includes a judicious mix of lineage-defining and/or immaturity markers, and has the ability to isolate stem cells, myeloblasts, monoblasts, immature erythroids, and immature lymphoid precursors (hematogones). Additional antibodies cover the complete normal maturation patterns for granulocytes, monocytes, and erythroids, as well as the identification of mature hematopoietic elements, such as mast cells, plasma cells, basophils, plasmacytoid dendritic cells, B cells, T cells, and NK cells.

For patients diagnosed at our institution, we used a similar lyse–wash–stain procedure, and a combination of antibodies including CD2, CD3, CD7, CD10, CD13, CD15, CD16, CD19, CD20, CD22, CD33, CD34, CD36, CD38, CD45, CD56, CD64, CD79a, CD117, HLA-DR, MPO, and Tdt, for non-MRD immunophenotyping.

To ensure the consistency of fluorescence intensity we employ a standardized workflow for the daily operation and periodic maintenance of the FACSLyric cytometers, based on manufacturer recommendations. The FACSLyric uses Cytometer Setup and Tracking (CS&T) beads to measure key performance factors such as linearity, detector efficiency, optical background, and electronic noise. Deviations in the key performance factors are then tracked and monitored over time. Daily QC includes performance quality control (PQC) and assay/tube settings setup (ATSS). Specifically, PQC will test and track the daily operation of the cytometer as compared to the cytometer’s characterization, while ATSS will adjust and update target tube values (TTVs), unique to each assay, and update assay compensation. Library settings for lyse/wash or lyse/no wash can be used to define assays. Alternatively, user-defined settings can be created for individual assays. Each assay saved on the individual FACSLyric cytometers has a standardized TTV that allows for standardization across all FACSLyric instruments in our laboratory. The user simply imports the desired assay onto the new cytometer and after PQC and ATSS the MFI will be standardized. Spillover values can be controlled entirely by the software in the FACSuite library or can be controlled entirely by the operator in a user-defined setting. Daily adjustments to ATSS will adjust compensation accordingly. A human cell check is performed to confirm that both the channel mean fluorescence intensity (MFI) and compensation are working correctly in identifying lymphocytes stained with CD3 fluorochromes (FITC, PE, PerCPcy5.5, PE-CY7, APC, Alexa-700, APC-H7, V450, V500, BV605, BV711 (12-color), BV786 (12-color). Weekly, monthly, three-month, and semi-annual maintenance and quality control monitoring are performed on each FACSLyric to ensure proper machine function and accurate patient results. Furthermore, there were no changes in methods, reagents, or equipment during the study period.

FC data files were analyzed using cluster-based algorithms in BD Infinicyt^TM^ 2.0 Software (BD, Franklin Lakes, NJ; Infinicyt™ is a trademark or registered trademark of Cytognos, S.L in Europe). Immunophenotypically abnormal myeloblasts were identified based on aberrant antigen expression, as previously described [26]. Briefly, leukemic blast populations were detected as distinct clusters in the MFC space based on light scatter properties (forward and side scatter), and the staining intensity of specific myeloblast-associated markers including, but not limited to, CD34, CD13, HLA-DR, CD117, CD123, CD33, CD371, and CD45. For reporting purposes, antigen expression on distinct clusters was designated as positive if at least 50% of the leukemic blast events overlapped with a normal internal control expressing those antigens. Antigens expressed below that threshold were recorded as negative. Bright (overexpressed) or dim (underexpressed) antigen expression was arbitrarily defined as a >0.5 log increase or decrease, respectively, in fluorescence intensity when compared to a normal counterpart (internal control). Whenever possible, normal precursor populations present in the AML sample were used as internal controls. Similar approaches have been described in the literature, albeit with varying cut-offs, depending on the individual clinical settings [27,28,29,30,31]. A change in immunophenotype was recorded for each antigen as either the loss or gain of aberrancy.

### 2.3. Statistical Analysis

Statistical analyses were carried out in GraphPad Prism, version 10.0 (GraphPad Software, San Diego, CA, USA). A statistically significant p value was considered as less than 0.05. Overall survival was studied using Kaplan–Meier analysis, with the log-rank (Mantel–Cox) test for curve comparison, and log-rank hazard ratios.

## 3. Results

### 3.1. Patient Characteristics

Our study included 61 women and 65 men, aged 19–87 (median, 63), diagnosed with AML. The respective AML diagnostic subcategories, as defined by the 2022 WHO classification, are listed in Table 2. Diagnostic material from patients initially diagnosed and/or treated at referring institutions, was reviewed at Mayo Clinic, Rochester, MN. Therapeutic protocols included induction chemotherapy, consolidation, allogeneic stem cell transplantation, maintenance, or re-induction, with regimens including, but not limited to, “7 + 3” (cytarabine + daunorubicin/idarubicin), hypomethylating agents (decitabine, azacitidine), venetoclax, HiDAC (high-dose cytarabine), FLAG-IDA (fludarabine, cytarabine, idarubicin, and granulocyte-colony stimulating factor), MEC (mitoxantrone, etoposide, cytarabine), midostaurin, gilterinib, sorafenib, cladribine, and pevonedistat. Bone marrow biopsies to evaluate treatment response and disease relapse were performed at time points according to established professional and institutional guidelines. Morphology, immunohistochemistry, and other ancillary testing, including chromosome and FISH analysis, MRD analysis by MFC and/or molecular testing, chimerism, and NGS were also applied according to a consensus algorithmic approach. For MRD testing, local guidelines were developed based on the 2021 consensus recommendations on AML MRD evaluation, developed by the ELN MRD Working Party [23]. Specifically, in patients undergoing intensive chemotherapy, the earliest time point for MRD assessment was obtained following count recovery after the first cycle of induction chemotherapy and no earlier than day +28. Furthermore, in patients not undergoing transplants, MRD was then obtained following count recovery at the end of consolidation therapy. Assuming continued complete remission, MRD assessment was then considered every 3 months for 2 years (or longer if clinically indicated) using both peripheral blood and bone marrow samples when available. Since our MRD guidelines were developed for both MFC and molecular assessment, peripheral blood is currently an acceptable specimen type only for molecular MRD testing. In transplant-eligible patients, MFC MRD was obtained at the pre-transplant evaluation, as well as post transplant on day +100 (in bone marrow) and, assuming continued complete remission, MRD assessment was considered every 3 months for 2 years (or longer if clinically indicated). In our cohort, MRD analysis by MFC was performed 1–66 months after diagnosis (median, 13); in 200/385 instances (52%), assessment was performed after an allogeneic stem cell transplant. Median follow-up was 38 months (range, 1–76) with 1–9 AML MRD flow analyses per patient (median, 3). At the most recent follow-up, 90/126 (71%) patients were alive.

### 3.2. AML MRD Flow Cytometry Assay Design and Performance

**Figure 1:**
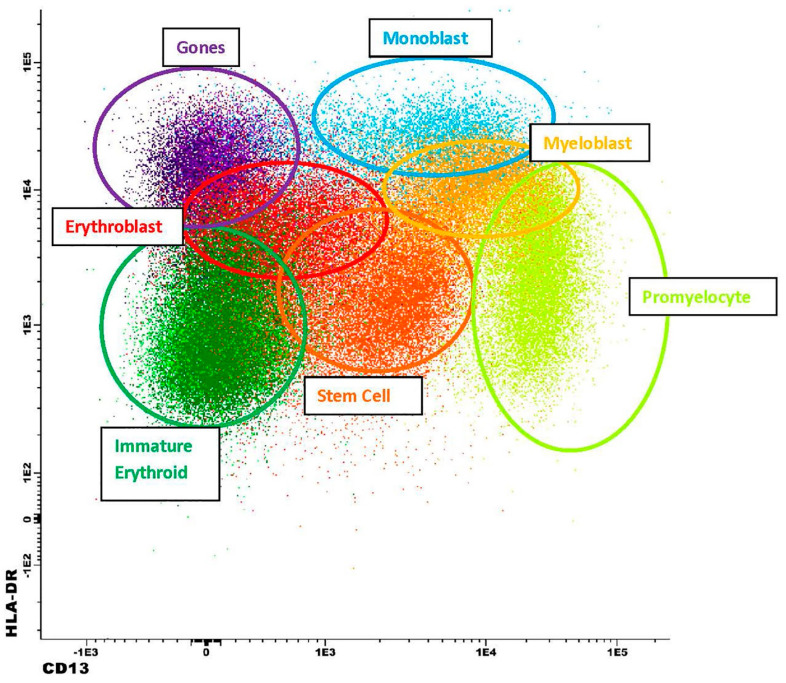
Typical immunophenotypic findings in a bone marrow aspirate collected from a normal control patient. CD13/HLA-DR expression pattern is shown in stem cells (orange), myeloblasts (dark yellow), promyelocytes (neon green), monoblasts (light blue), erythroblasts (red), hematogones (purple), immature erythroid precursors (beyond the pronormoblast maturation stage; green), respectively.

**Figure 2:**
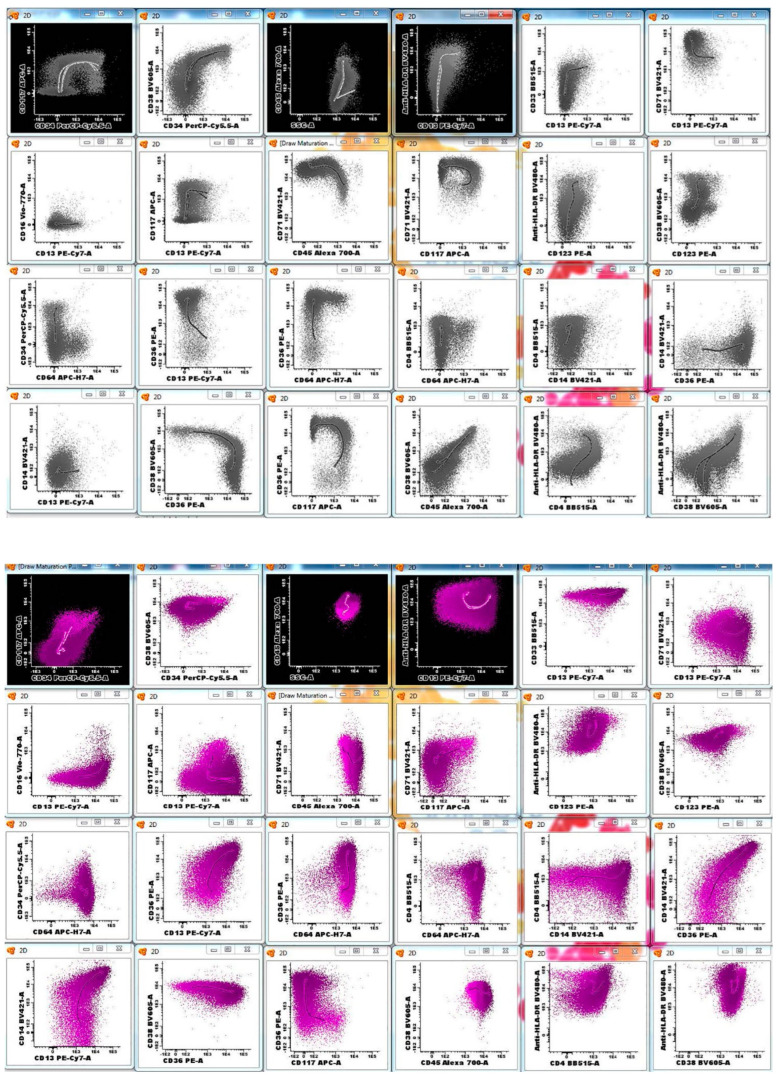

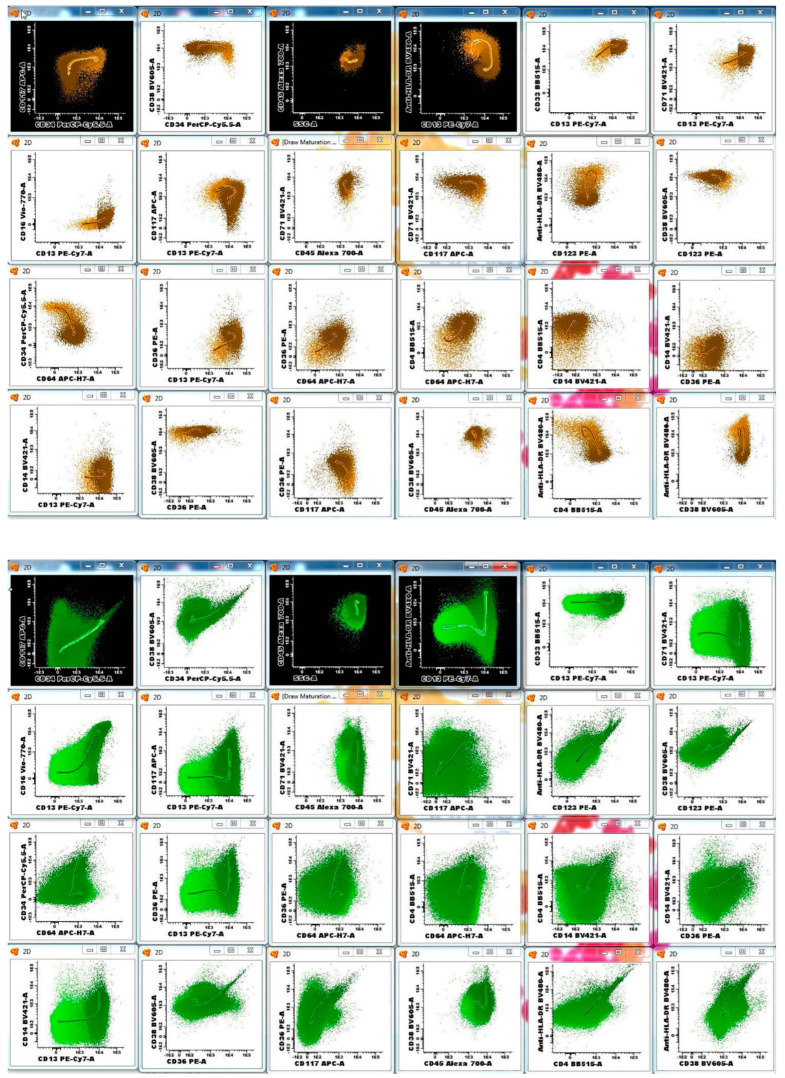
Typical immunophenotypic findings in a bone marrow aspirate collected from a normal control patient. Common antigen expression and maturation patterns are shown for erythroid precursors (gray), monocytic precursors (purple), early myeloid precursors (brown), and late myeloid precursors (green).

The typical antigen expression in normal cell lineages with this antibody panel is shown in Table 3. These data were derived from the initial validation set of 25 normal/reactive control bone marrows and subsequently confirmed in 200 additional cases. For example, in a non-MRD iteration of a precursor antibody panel, CD13 and HLA-DR expression patterns have been extensively evaluated in our laboratory on CD34-positive myeloblasts, in normal controls, and in individuals with myeloid neoplasms [32]. These patterns were then used to identify abnormal maturation curves in patients with clonal cytopenias of uncertain significance (CCUS) and myelodysplastic syndrome, respectively [26]. Similarly, these and other antigens were applied in normal controls or patients with reactive/regenerative bone marrows (and without a known history of chronic or acute myeloid neoplasms) and consistent, reproducible maturation patterns were identified for normal lineages. Examples are shown in Figure 1 and Figure 2. In this analysis, myeloid blasts exhibit a well-defined spectrum of antigen expression as they mature. Starting with the stem cells, which are dim positive for CD13, CD33, and HLA-DR, and negative to dim positive for CD38, the myeloid blasts then gain normal levels of CD13, CD33, HLA-DR, and CD38. At the same time, both stem cells and myeloblasts are positive for CD34, CD117, and dim positive for CD45 and CD123. CD71 is typically negative in stem cells and dim positive in myeloblasts. Finally, CD371 is uniformly expressed in myeloid blasts, while being negative in stem cells. Normal myeloblasts are usually negative for CD16, CD36, CD61, CD14, CD4, CD56, CD15, CD7, and CD19.

For analyte and matrix stability, we evaluated 18 bone marrow specimens (for a total of 90 replicates) shipped and stored at ambient temperature. Samples from three normal controls (waste bone marrow from an orthopedic hip replacement or from negative lymphoma-staging bone marrow biopsies) and three patients with a known diagnosis of AML, collected in ACD, EDTA, and heparin, respectively, were tested at baseline (on the day of specimen collection), and then at 24, 48, 72, and 96 h after collection. The results from accepted time points had to be qualitatively the same (MRD-positive or -negative, based on the validated cut-off value of 0.01%) and if MRD-positive, the semi-quantitative value should have a coefficient of variation (CV) <30%. MRD values <1.0% may have a CV > 30% and still be acceptable as long as they are qualitatively the same as the baseline sample. The data show that MRD detection can be confidently assessed up to 96 h post draw. However, according to CLSI-H62 recommended practices, the last acceptable time point was defined as the one prior to the latest test that also met the passing criteria. Accordingly, the stability for this assay is defined as 0–72 h (validated to the day, not the hour). Similar results were obtained for matrix equivalence, when comparing results obtained from individual samples, regardless of the anticoagulant (ACD, EDTA, or heparin) the bone marrow sample was collected in. Although no cell viability marker is included in the antibody panel, our analysis strategy is designed to ensure that the most relevant, highest quality acquired events are included in the analysis, by removing doublets/aggregates, debris, non-viable cells, and electronic noise, based on light scatter properties, forward scatter vs. time, and forward scatter amplitude vs. height, as recommended by the ELN guidelines [21,23].

Precision/reproducibility was evaluated on three abnormal specimens, containing around 0.1% of abnormal blasts. For intra-assay reproducibility, each abnormal sample was set up in triplicate and had to yield qualitatively similar results (MRD-positive) with a CV < 20% for all replicates. Similarly, inter-assay reproducibility was verified on three abnormal bone marrow specimens, for a total of nine replicates, set up and analyzed by multiple operators, and assessed in runs performed at different times. Each replicate had to show qualitatively similar results (MRD-positive) and at a CV < 20%. This standardized approach, including uniform instrument setting and gating strategy, showed a precise assay near the assay’s limit of detection.

For accuracy we performed split-sample analysis on 32 bone marrow specimens with the AML MRD flow cytometry assay developed by the University of Washington [33]. The results showed 100% concordance between the two modalities in 29/32 (91%) of specimens that were either MRD-negative or showed MRD ≥ 0.1%. Three additional samples were found to be positive for MRD by our assay; two of them showed immunophenotypically aberrant blasts between 0.01 and 0.09%, while one showed MRD ≥ 0.1%. The presence of residual disease was confirmed on all three of these specimens by additional ancillary testing (cytogenetics/FISH and/or molecular analysis) showing abnormal results. Given the higher sensitivity of our assay, these accuracy results meet the limitations of the closeness of agreement recognized for this type of assay [34].

Reportable range/linearity was established from 61 bone marrow samples (25 normal and 33 patients). The 25 normal specimens were negative for MRD and showed zero abnormal events (no immunophenotypically aberrant blasts detected). The 33 specimens from patients with AML had 11 cases that were MRD-positive and 22 that were MRD-negative. The positive cases showed a range of 0.01–86% of aberrant blasts. Linearity was tested in four serial dilutions from three samples (at 1%, 0.1%, 0.01%, and 0.001%, respectively). The results show that the assay produces linear results at validated MRD detection levels (R^2^ = 0.9964).

To verify analytical sensitivity, we spiked three bone marrow samples from patients with AML into normal bone marrow to achieve a calculated tumor burden of 1% (baseline), 0.1%, 0.01%, and 0.005% (half-log below the assay’s limit of detection, based on previous guidance [25]). All three samples showed a precise MRD-positive limit of detection at 0.01%. Of note, the lower limit of quantification (LOQ) is defined by an abnormal population (aberrant blasts) of at least 50 events, detected in each of the three tubes. This translates into a minimum of 500,000 events collected per tube for reporting MRD results without a qualifier for low event collection. However, we routinely collect at least 1,000,000 analyzed events per tube (typically, 1,200,000); hence, the theoretical lower limit of quantification (LOQ) of 0.005%. The lower limit of detection (LOD) is defined as a cluster of at least 20 events with distinct immunophenotype. Furthermore, the limit of blank for this assay was not determined based on a single gate or two-dimensional plot, but rather by the same multi-parameter Boolean analysis strategy used to identify abnormal clusters of aberrant blasts. When applying all 12 parameters (10 unique antigens plus light scatter parameters) in three tubes, the limit of blank for this assay is zero. Furthermore, as the gating strategy is designed to identify abnormal clusters of blasts composed of at least 50 events, with distinct immunophenotypic characteristics (LOQ), any “abnormal” cell population with fewer than 50 events will not be considered as an indication of MRD positivity, regardless of the immunophenotype, for reporting purposes.

Analytic specificity was verified in 25 normal bone marrow specimens (waste bone marrow from orthopedic hip or shoulder replacement, or from negative lymphoma staging bone marrow biopsies). These samples were also used to develop an immunophenotypic fingerprint of normal/reactive myeloid blasts and used in the “DfN” analysis to identify aberrant myeloid blasts. Based on these 25 normal bone marrow samples, the specificity of our assay is 100%.

Additional data from assay validation are included in the Supplementary Materials (Tables S1–S5).

### 3.3. Immunophenotypic Findings in Patients with Positive AML MRD Flow Cytometry Analyses

**Figure 3:**
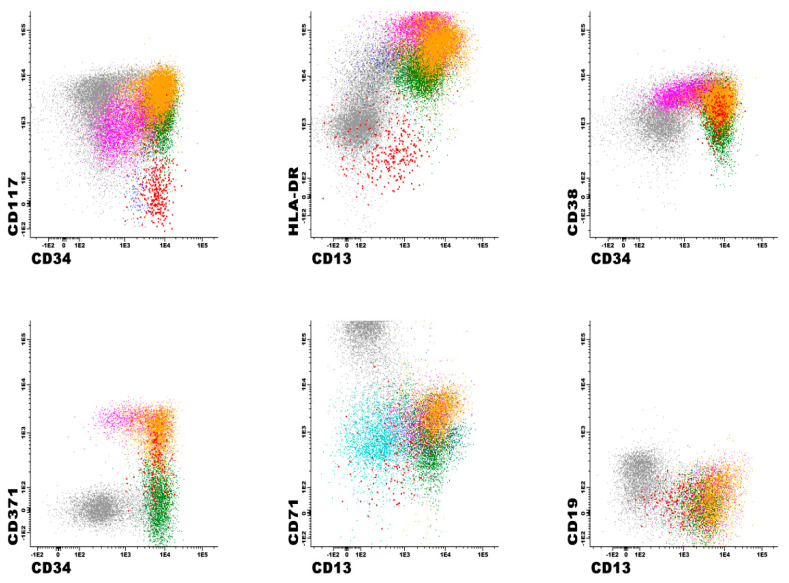

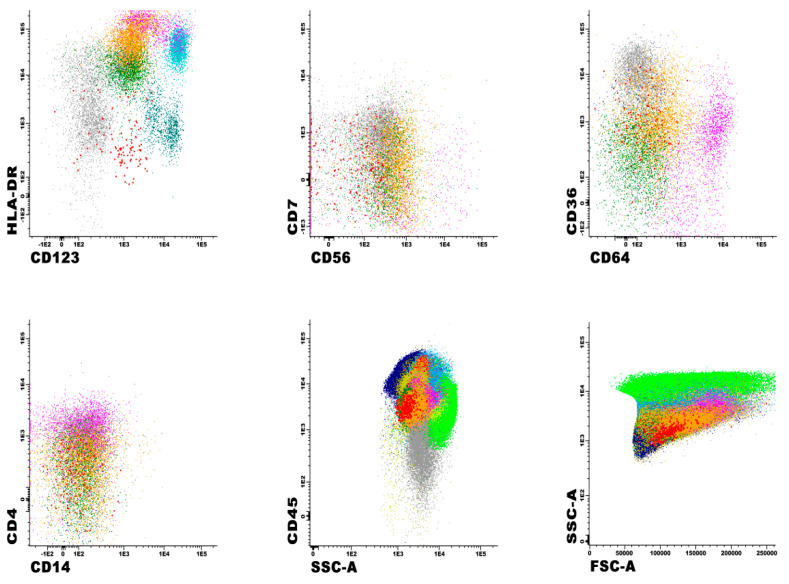
An example of an MRD-positive flow cytometry bone marrow sample collected from a patient with acute myeloid leukemia, status post chemotherapy. The neoplastic myeloblasts (red) show multiple immunophenotypic aberrancies, such as decreased expression of CD13, CD38, and CD371, and negative expression of CD117 and HLA-DR. Also present are normal myeloblasts (orange), promyelocytes (neon green), mature granulocytes (green), monoblasts (pink), basophils (dark green), plasmacytoid dendritic cells (cyan), and erythroids (gray).

AML MRD was detected by MFC in 32/126 (25%) patients and in 77/385 (20%) analyses. The median aberrant blast percentage in MRD-positive cases was 1.87% (range, 0.01–12), with 8/77 (10%) cases and 32/77 (42%) cases showing <0.1% and <1% abnormal myeloblasts, respectively. We achieved the median target of 1,200,000 events per tube in all cases. Low event acquisition occurred in 48/385 (13%) of cases, including 26/385 (7%) with detectable disease and 22/385 (6%) MRD-negative cases. When AML MRD was present, at least one of all 18 tested antigens showed aberrant expression in all cases (Table 4). These aberrancies include the overexpression, underexpression (including complete absence of expression), or asynchronous expression of antigens. Figure 3 shows an example with multiple immunophenotypic aberrancies, such as decreased CD13, CD38, and CD371, and negative CD117 and HLA-DR, compared to normal myeloblasts from the same specimen. Aberrancies ranged from 2 to 12 per case (median, 5), with CD36 expression being the most common (66%) (Table 4). Other frequent aberrancies included negative/underexpressed CD13 or CD371 (53%); negative CD34 (51%); positive CD56 (46%); negative/underexpressed CD123 (41%), CD117 (38%), and CD38 (33%); and positive CD7 (39%) (Table 4).

All follow-up cases demonstrated multiple aberrancies. In patients with serial positive MRD analyses, the immunophenotype remained similar to a previously established immunophenotypic fingerprint in 75% of cases (58/77). In 21% (16/77), the immunophenotype differed from baseline, while 4% (3/77) lacked a sufficiently characterized baseline for comparison. Changes in immunophenotype included CD34 (11/16, 69%); CD117 and CD371 (9/16, 56%, each); CD13 (8/16, 50%); CD7 (7/16, 44%); CD56 (6/16; 38%); HLA-DR (5/16, 31%); CD33 (4/16, 25%); CD4 and CD15 (3/16; 19%, each); and CD19 (2/16, 13%).

**Figure 4:**
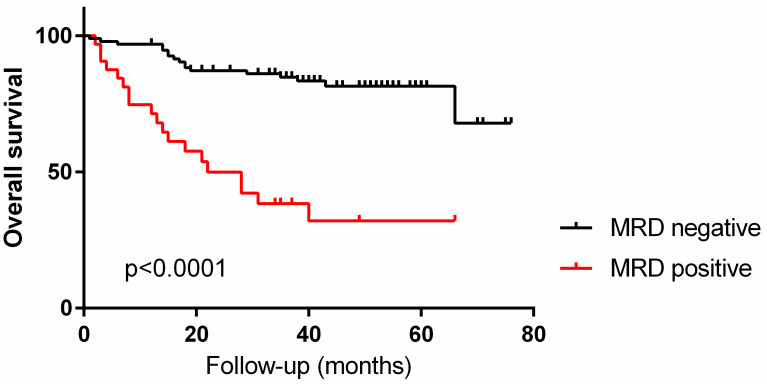
Overall survival in 126 patients with acute myeloid leukemia, based on MRD positivity status in first studied sample. Patients with MRD-positive samples are shown in red.

MRD positivity detected in the first sample studied was associated with unfavorable overall survival (hazard ratio: 5.153, 95% CI: 4.926–27.16, p < 0.0001) (Figure 4).

### 3.4. Correlation with Morphology and Other Ancillary Testing Findings

The bone marrow aspirate differential count revealed a median blast percentage of 3% in the 77 AML MRD-positive cases by MFC, with 75% (58/77) having <5% blasts, and 86% (66/77) showing <10% blasts. Our institutional guidelines allow for MRD assessment by MFC post therapy when blast percentages are <5% by morphology (i.e., in complete remission). However, hematopathologists can reflex to AML MRD flow testing for cases with 5–10% blasts, especially in markedly hypocellular bone marrows or in cases with challenging morphology. Notably, 10% (39/385) of total AML MRD flow analyses and 31% (24/77) of positive cases, had markedly hypocellular bone marrow biopsies (<5% cellularity).

Cytogenetic studies were performed and yielded successful results in 86% (66/77) of MRD-positive cases by MFC. Of these, 54% (27/50) had genetic abnormalities detected at diagnosis still present at the time of MRD evaluation. Similarly, 26% (7/27) with gene abnormalities detected by FISH at diagnosis, were also positive at the time of AML MRD identified by flow cytometry. Finally, 58% (11/19) of cases with a documented *NPM1* or *FLT3* mutation at diagnosis showed concordant findings (MRD AML) with MFC at follow-up (including 100% concordance for *NPM1* testing).

## 4. Discussion

Measurable residual disease (MRD) is an independent prognostic indicator in AML, providing valuable insights for risk stratification and treatment planning, alongside established clinical, cytogenetic, and molecular findings assessed at diagnosis [2,7,9,15]. The applications of MRD status determination in AML are multifaceted, including (1) establishing a deeper remission status; (2) refining outcome prediction and informing post-remission treatment; (3) identifying impending relapse and enabling early intervention; (4) enhancing post-transplant surveillance; and (5) serving as a surrogate end point for accelerated drug testing and approval [6,8,10,13,14,16,17]. MFC immunophenotyping for AML MRD is particularly useful post treatment and both before and after hematopoietic stem cell transplantation. Under ideal circumstances, the perfect MRD assay would precisely identify the lowest level of leukemic blasts in patients with AML in morphologic CR, which, if left untreated, would lead to relapse in all patients. At the same time, the ideal MRD assay would accurately distinguish these leukemic blasts from any other, immunophenotypically aberrant, and potentially similar (but not identical) precursor cells that do not cause relapse. With perhaps rare exceptions (e.g., molecular testing in acute promyelocytic leukemia), such an MRD test does not exist for AML patients as a group [5,7,8,14,17,18,19,33]. The current state is reflected in the well-known observation that a significant proportion of patients achieving MRD-negative status (by various modalities) may still relapse, while some MRD-positive patients will not. This variable behavior is likely a combination of the complex heterogeneity of biological determinants in AML, as well as different therapeutic regimens that are being applied.

In the absence of a perfect MRD test, various modalities such as MFC, real-time quantitative PCR, and NGS are employed for residual disease assessment in AML [2,5,10,22,23,34]. Each method has its advantages and limitations, and their applicability is guided by international expert recommendations [2,22,23,34]. MFC-based AML MRD methods, in particular, require significant expertise and capacity, limiting their availability to a few academic centers and reference laboratories in the United States. This situation is different in Europe, where many clinical hematology units are connected with a well-equipped ISO-certified MFC laboratory able to perform MRD studies in hematologic malignancies [14,18,23].

Our study presents preliminary findings from a sensitive and accurate AML MRD MFC assay, validated to be used as an additional powerful tool in the armamentarium of current decision-making modalities for AML risk stratification, prognosis, and therapy. This assay meets and often exceeds the ELN AML MRD Working Party recommendations, developed in 2018 and updated in 2021 [21,22,34], including, but no limited to: (1) availability of a diagnostic sample; (2) implementation of a minimum required set of antibody combination to ensure harmonized LAIP/DfN MRD detection, analysis, and reporting; (3) uniform use of the LAIP/DfN strategy for MRD detection that incorporates recommended core MRD markers (CD7, CD13, CD33, CD34, CD45, CD56, CD117, and HLA-DR); (4) evaluation of specificity of immunophenotypic blast aberrancies in control specimens (including regenerating bone marrows); (5) using “first-pull” bone marrow aspirate for MRD assessment, and processing it within 72 h; (6) clinical-grade MRD assay validation, based on the guidelines for rare-event detection by flow cytometry; (7) the use of a gating strategy including FSC vs. time and doublet exclusion plots to ensure the highest quality of relevant acquired events; (7) the acquisition of at least 500,000 CD45-positive cells and ≥100 viable cells in the “blast gate” as a threshold for flow cytometric MRD negativity; and (8) the assessment of MRD assay performance based on a calculated lower limit of detection (LOD) and lower limit of quantification (LOQ) [21,23].

The assay’s sensitivity (LOQ) is conservatively validated at 0.01% when routinely evaluating 1,000,000 events per tube (to reflect the minimum requirement of 500,000 events collected per tube). In our cohort of 126 patients and 385 analyses, we were able to acquire a median number of 1,200,000 events per tube, with only 6–7% of cases showing low-event acquisition (at least one tube with less than 500,000 events) and a similar percentage in both MRD-positive and MRD-negative cases. This known potential downside of AML MRD evaluation by MFC may be in part due to our institutional guidelines of MRD assessment, which include earlier time points (i.e., after the first cycle of induction/therapy) and allow for flexibility in the “count recovery” criterion from the ELN recommendations [23].

As noted in the literature, and applicable to other hematologic malignancies (such as multiple myeloma, B lymphoblastic leukemia, and chronic lymphocytic leukemia), the concept of a lower limit of detection and a lower limit of quantification in rare-event analysis by MFC also represents a crucial advancement in MRD studies in AML [20,21,23,24,25,34]. For our MFC AML MRD assay, we adopted common cut-offs used by other laboratories for LOD (set at 20 events and indicating the threshold below which the test should be considered negative) and LOQ (set at ≥50 events to define MRD-positive status for clinical reporting purposes). For clinical reporting, we also adopted the consensus guidelines that recommend using 0.1% as the threshold to distinguish MRD-positive from MRD-negative patients. However, MRD levels below 0.1% may be consistent with residual AML, and several studies have shown the prognostic significance of MRD values below 0.1% [10,15,34,35,36]. In that respect, it is likely that the assessment of MRD positivity on a sliding scale (rather than specific cut-offs) would yield valuable prognostic information. There exist data that suggest that the gray zone between LOD and LOQ may provide further insight into the actual MRD status and may also retain some prognostic significance [35]. We may be able to observe similar findings in our patient population once we complete further accrual and longitudinal data analysis. This situation highlights another question, namely whether the concept that a predefined AML MRD threshold level (either 0.1% or lower) may fit all cases. As shown in recent studies [36,37], it seems that not only the AML subtype, but also the therapeutic protocol, the number of therapy cycles, and the patient’s age, may require more specific and dedicated MRD thresholds, not to mention the choice of sampling timepoints. In our patient cohort, it is difficult to draw specific conclusions based on these variables, due to the heterogeneity of the current study group. However, we intend to further study this hypothesis once we complete further accrual and more detailed longitudinal data analysis.

Our assay benefits from a broad antibody combination, allowing for the detection of blast aberrancies even in the absence of a diagnostic immunophenotype. While 96% of our patients had a sufficiently detailed baseline flow to establish a diagnostic LAIP, the number and range of antibodies used enabled us to pursue an integrated LAIP-based DfN approach, as recommended by the ELN 2021 AML MRD Working Party [23], and that is applicable even for patients in which a diagnostic immunophenotype is not accessible. Similarly, variations in immunophenotypes are well known and can occur due to the expansion or development of subclones after therapy. For these, an analytic strategy based on LAIP alone is insufficiently sensitive to capture MRD-positive cases accurately. We have documented immunophenotypic changes in 21% of the MRD-positive cases that were still aberrant enough for our assay to both identify these abnormal populations accurately and to correctly separate them from non-neoplastic precursor cell aberrancies. This fact is also supported by the concordance with other ancillary testing (such as cytogenetics/FISH and molecular analysis, when performed) in patients with positive MRD MFC, and by the known deleterious impact of MRD positivity on patient outcomes [6,9,15,19]. In our patient cohort, MRD positivity detected in the first sample studies was associated with a 5-year OS of 32%, compared to 68% in the MRD-negative patients, similar to other studies [6,15].

The rate of MFC MRD positivity in our study was 25% for individual patients, and 20% for all analyzed, reflecting a higher proportion of MRD-negative follow-up cases. These results support the use of MRD testing primarily in bone marrows with documented CR, as 75% of MRD-positive flow cases occurred in samples with less than 5% blasts by morphology. Our institutional guidelines allow for some flexibility in performing MRD analysis in cases with higher morphologic blast percentages (5–10%), accounting for biological variations such as hypocellular bone marrows and reactive expansions of normal (non-leukemic) blasts. By broadening this criterion, we were still able to limit the majority of MRD-positive MFC cases (86%) to bone marrow samples with <10% blasts by morphology. Currently, this approach facilitates MRD assessment for relevant prognostic information, while preserving sufficient bandwidth in our flow cytometry laboratory to offer this (still) resource-intensive assay.

The latter observation highlights one of the main obstacles that currently limits the widespread use of AML MRD MFC, which is the typical requirement of lengthy manual analysis time, due to the complex maturation background found in normal and leukemic cell lineages. Median analysis times of 45–60 min for our assay limit the number of specimens that can be consistently analyzed. The issue is confounded by the requirement of lengthy training times for laboratory technologists to achieve the requisite expertise, and challenging reimbursement policies, while balancing routine immunophenotyping analyses, particularly in a busy reference laboratory. One potential solution for this problem is the development of automated analysis strategies, that have the potential to drastically reduce analytic times while maintaining accuracy and sensitivity [19,38,39].

## 5. Conclusions

In summary, we describe our experience with an accurate and sensitive MFC assay for the assessment of MRD in a large series of patients with AML. Despite current limitations in the standardization and widespread deployment of this modality in routine clinical practice, we demonstrate the feasibility of a robust validation protocol (including stability, precision, accuracy, sensitivity, specificity, and linearity) to generate an assay that is capable of becoming a valuable addition to current decision-making tools for AML management.

## Figures and Tables

**Table 1:** Antibody composition for acute myeloid leukemia (AML) measurable residual disease (MRD) analysis by flow cytometry.

		FITC	PE	PerCP or PerCP-Cy5.5	PE-Cy7	APC	APC-H7	V450	V500	APC-R700	BV605
Tube 1	Antibody	CD33 BB515	CD123	CD34	CD13	CD117	CD16 APC-Vio770	CD71 BV421	HLA-DR BV480	CD45 APC-AF700	CD38 BV605
	Catalog#	564588	306006	347213	338432	341106	130-113-393	562995	566113	560566	562665
	Vendor	BD Horizon	BioLegend	BD	BD	BD	Miltenyi Biotec	BD Horizon	BD Horizon	BD Pharmingen	BD Horizon
Tube 2	Antibody	CD4 BB515	CD36	CD34	CD13	CD117	CD64 APC-Fire750	CD14 BV421	HLA-DR BV480	CD45 APC-AF700	CD38 BV605
	Catalog#	564419	555455	347213	338432	341106	305036	659450	566113	560566	562665
	Vendor	BD Horizon	BD Pharmingen	BD	BD	BD	BioLegend	BD Horizon	BD Horizon	BD Pharmingen	BD Horizon
Tube 3	Antibody	CD7 BB515	CD19	CD34	CD13	CD117	CD371 APC-Vio770	CD56 BV421	HLA-DR BV480	CD45 APC-AF700	CD15 BV605
	Catalog#	565211	340720	347213	338432	341106	130-106-485	562751	566113	560566	562980
	Vendor	BD Horizon	BD	BD	BD	BD	Miltenyi Biotec	BD Horizon	BD Horizon	BD Pharmingen	BD Horizon

**Table 2:** Acute myeloid leukemia (AML) diagnoses in the study cohort, according to the WHO 2022 classification.

AML Subtype	Count	Percentage
AML, myelodysplasia-related	33/126	26%
AML with NPM1 mutation	31/126	25%
AML with biallelic TP53 inactivation	11/126	9%
AML post cytotoxic therapy	10/126	8%
AML with CEBPA mutation	4/126	3%
AML with CBFB::MYH11 fusion	4/126	3%
AML with KMT2A rearrangement	4/126	3%
AML with RUNX1::RUNX1T1	3/126	3%
AML, transformation from a prior chronic myeloid neoplasm	3/126	3%
AML with DEK::NUP214 fusion	2/126	2%
AML with NUP98 rearrangement	2/126	2%
Myeloid sarcoma	2/126	2%
Mixed phenotype acute leukemia, B/myeloid	2/126	2%
Mixed phenotype acute leukemia, T/myeloid	1/126	1%
AML defined by differentiation	12/126	11%
AML with maturation	4/126	3%
Acute monocytic leukemia	2/126	2%
Acute myelomonocytic leukemia	6/126	5%

**Table 3:** Typical antigen expression in normal cell lineages with the acute myeloid leukemia measurable residual disease antibody panel. Antigen expression is listed as positive (“+”); dim positive (“dim”), defined as 0.5 log lower intensity compared to normal internal control (blasts or lymphocytes, as applicable); subset positive (“+/−”); bright positive, defined as a 0.5–1 log higher intensity compared to normal internal control (blasts or lymphocytes, as applicable); and negative (“−”).

	Stem Cells	Myeloblasts	Monoblasts	Erythroids (Immature)	Erythroids (Maturing)	Basophils	PDCs	Promyelocytes	Hematogones (“Mature”)	Hematogones (“Immature”)	Plasma Cells
CD34	+	+	+/−	+	−	−	−	−	−	+	−
CD117	+	+	+	+	+	−	−	+	−	−	−
HLA-DR	dim	+	bright	dim	dim	−	+	+	+	+	dim
CD13	dim	+	+	−	−	+	−	bright	−	−	−
CD45	dim	dim	dim	dim	dim	+	+	dim	dim	dim	dim
CD38	−/dim	+		+	dim	bright	+	+	bright	bright	bright
CD123	dim	dim	+	−	−	bright	bright	−	+/−	+/−	−
CD71	−	dim	dim	dim	bright	−	−	dim	−	−	−
CD33	dim	+	+	−	−	+	−	+	−	−	−
CD16	−	−	−	−	−	−	−	−	−	−	−
CD36	−	−	−	dim	bright	+	+	−	−	−	−
CD64	−	−	dim	−	−	−	−	−	−	−	−
CD14	−	−	−	−	−	−	−	−	−	−	−
CD4	−	−	dim	−	−	−	+	−	−	−	−
CD371	−	+	+	−	−	dim	+/−	+	−	−	−
CD56	−	−	−	−	−	−	+/−	−	−	−	+/−
CD15	−	−	−	−	−	−	−	+/−	−	−	−
CD7	−	−	−	−	−	−	+/−	−	−	−	−
CD19	−	−	−	−	−	−	−	−	+	+	+/−
	**B Cells**	**T Cells**	**NK Cells**		**Mature Monocytes**		**Promonocytes**		**Mast Cells**	**Eosinophils**	
CD34	−	−	−		−		−		−	−	
CD117	−	−	−		−		+/−		bright	−	
HLA-DR	bright	+/−	−		+		bright		dim	+	
CD13	−	−	−		+		+		−/dim	+	
CD45	+	+	+		+		+		dim	+	
CD38	+/−	−	+		+		+		−	+	
CD123	−	−	−		dim		+		−	−	
CD71	−	−	−		−		−		dim	+	
CD33	−	−	−		+		+		+	+	
CD16	−	−	+		+/−		−		−	−	
CD36	−	−	−		+		−		−	dim	
CD64	−	−	−		+		+		−	−	
CD14	−	−	−		+		−		−	−	
CD4	−	+/−	−		dim		dim		dim	−	
CD371	−	−	−		+		+		+	+	
CD56	−	+/−	+		−		−		−	−	
CD15	−	−	−		+/−		+/−		−	−	
CD7	−	+	+		−		−		−	−	
CD19	+	−	−		−		−		−	−	

**Table 4:** Aberrant expression of antigens on myeloid blasts in patients with measurable residual acute myeloid leukemia.

Aberrant Antigen Expression	Abnormal	Dim/Negative	Bright
CD36 (positive)	51/77 (66%)		
CD13	45/77 (58%)	41/77 (53%)	4/77 (5%)
CD371	43/77 (56%)	41/77 (53%)	2/77 (3%)
CD34 (negative)	39/77 (51%)	33/77 (43%)	6/77 (8%)
CD56 (positive)	35/77 (46%)		
HLA-DR	35/77 (46%)	30/77 (39%)	5/77 (7%)
CD123	32/77 (42%)	31/77 (41%)	1/77 (1%)
CD7 (positive)	30/77 (39%)		
CD117	30/77 (39%)	29/77 (38%)	1/77 (1%)
CD38	27/77 (36%)	25/77 (33%)	2/77 (3%)
CD33	25/77 (33%)	13/77 (17%)	12/77 (16%)
CD15 (positive)	17/77 (21%)		
CD64 (positive)	14/77 (18%)		
CD4 (positive)	13/77 (17%)		
CD19 (positive)	6/77 (8%)		
CD45 (positive)	5/77 (7%)		

## Data Availability

The original contributions presented in this study are included in the article/Supplementary Materials. Further inquiries can be directed to the corresponding author.

## Supplementary Materials

The following supporting information can be downloaded at: https://www.mdpi.com/article/10.3390/cancers17071155/s1, Table S1: Data for stability studies from 9 abnormal bone marrow samples collected in ACD, EDTA, and heparin; Table S2: Precision/reproducibility studies: data for intra-assay reproducibility from 3 abnormal bone marrow samples; Table S3: Precision/reproducibility studies: data for inter-assay reproducibility from 3 abnormal bone marrow samples; Table S4: Accuracy comparison of our flow cytometry assay (AMLMD) with the AML MRD assay at the University of Washington (UW) (split sample analysis). Discordant results are highlighted; Table S5: Analytic sensitivity/limit of detection—serial dilution data. The first serial dilution targeted a blast percentage of 1%, followed by three 1:10 dilutions after that.
