# Supplementary material for: Measurable Residual Disease Analysis by Flow Cytometry: Assay Validation and Characterization of 385 Consecutive Cases of Acute Myeloid Leukemia
Source: Cancers (Basel). 2025 Mar 29;17(7):1155. doi: 10.3390/cancers17071155 (PMC11987847; doi:10.3390/cancers17071155)
Supplement: Supplementary file 1 [file cancers-17-01155-s001.zip › cancers-3480351-supplementary.pdf]

Supplementary Materials: Measurable Residual Disease Analysis by Flow Cytometry: Assay Validation and Characterization of 385 Consecutive Cases of Acute Myeloid Leukemia

Husam Jum'ah, Gregory E. Otteson, Michael M. Timm, Matthew J. Weybright, Min Shi, Pedro Horna, Dragan Jevremovic, Kaaren K. Reichard and Horatiu Olteanu

Table S1. Data for stability studies from 9 abnormal bone marrow samples collected in ACD, EDTA, and heparin.

Sample	%MRD	QC Pass
**Stability-1-Baseline**	**0.1652**	
Stability-1-24hr	NA	-
Stability-1-48hr	0.1751	Yes
Stability-1-72hr	0.2073	Yes
Stability-1-96hr	0.2385	Yes
Mean	0.1965	
SD	0.0333	
% CV	16.9	
**Stability-2-Baseline**	**0.3376**	
Stability-2-24hr	NA	-
Stability-2-48hr	0.3391	Yes
Stability-2-72hr	0.3384	Yes
Stability-2-96hr	0.3347	Yes
Mean	0.3375	
SD	0.0019	
% CV	0.57	
**Stability-3-Baseline**	**0.2069**	
Stability-3-24hr	0.2028	Yes
Stability-3-48hr	0.2094	Yes
Stability-3-72hr	0.2227	Yes
Stability-3-96hr	0.2283	Yes
Mean	0.2140	
SD	0.0109	
% CV	5.1	
**Stability-4-Baseline**	**0.4514**	
Stability-4-24hr	NA	-
Stability-4-48hr	0.4785	Yes
Stability-4-72hr	0.4618	Yes
Stability-4-96hr	0.4417	Yes
Mean	0.4583	
SD	0.0157	
% CV	3.4	
**Stability-5-Baseline**	**0.0231**	
Stability-4-24hr	0.0102	Yes
Stability-4-48hr	0.0214	Yes
Stability-4-72hr	0.0173	Yes
Stability-4-96hr	0.0272	Yes

Mean	0.1984	
SD	0.0065	
% CV	32.5	
**Stability-6-Baseline**	**0.4047**	
Stability-4-24hr	0.5165	Yes
Stability-4-48hr	0.4768	Yes
Stability-4-72hr	0.5794	Yes
Stability-4-96hr	0.4960	Yes
Mean	0.4947	
SD	0.0634	
% CV	12.8	
**Stability-7-Baseline**	**0.1352**	
Stability-4-24hr	0.1519	Yes
Stability-4-48hr	0.1309	Yes
Stability-4-72hr	0.0896	Yes
Stability-4-96hr	NA	-
Mean	0.1269	
SD	0.0265	
% CV	20.9	
**Stability-8-Baseline**	**3.9519**	
Stability-4-24hr	3.4546	Yes
Stability-4-48hr	3.5216	Yes
Stability-4-72hr	4.4427	Yes
Stability-4-96hr	4.8330	Yes
Mean	4.0408	
SD	0.5938	
% CV	14.7	
**Stability-9-Baseline**	**5.4330**	
Stability-4-24hr	6.0516	Yes
Stability-4-48hr	5.8128	Yes
Stability-4-72hr	5.8450	Yes
Stability-4-96hr	6.1217	Yes
Mean	5.8548	
SD	0.2652	
% CV	4.5	

Table S2. Precision/reproducibility studies: data for intra-assay reproducibility from 3 abnormal bone marrow samples.

Sample	%MRD	QC Pass
**Sample 1**		
Intra-assay-1a	0.5914	Yes
Intra-assay-1b	0.5890	Yes
Intra-assay-1c	0.5291	Yes
Mean	0.5699	
SD	0.0353	
% CV	6.2	
**Sample 2**		
Intra-assay-2a	0.3363	Yes
Intra-assay-2b	0.3841	Yes
Intra-assay-2c	0.3819	Yes
Mean	0.3674	

SD	0.0270	
% CV	7.3	
**Sample 3**		
Intra-assay-3a	0.1716	Yes
Intra-assay-3b	0.1496	Yes
Intra-assay-3c	0.1466	Yes
Mean	0.1559	
SD	0.0137	
% CV	8.8	

Table S3. Precision/reproducibility studies: data for inter-assay reproducibility from 3 abnormal bone marrow samples.

Sample	%MRD	QC Pass
**Sample 1**		
Inter-assay-1a	0.5914	Yes
Inter-assay-1b	0.5890	Yes
Inter-assay-1c	0.5291	Yes
Mean	0.5575	
SD	0.0247	
% CV	4.3	
**Sample 2**		
Inter-assay-2a	0.3384	Yes
Inter-assay-2b	0.3380	Yes
Inter-assay-2c	0.3363	Yes
Mean	0.3376	
SD	0.0011	
% CV	0.3	
**Sample 3**		
Inter-assay-3a	0.1441	Yes
Inter-assay-3b	0.1716	Yes
Inter-assay-3c	0.1666	Yes
Mean	0.1608	
SD	0.0146	
% CV	9.1	

Table S4. Accuracy comparison of our flow cytometry assay (AMLMD) with the AML MRD assay at the University of Washington (UW) (split sample analysis). Discordant results are highlighted.

Sample	AMLMD MRD result	AMLMD MRD (%)	UW MRD result	UW MRD (%)
Accuracy-1	Positive	1.3766	Positive	0.7
Accuracy-2	Negative	-	Negative	-
Accuracy-3	Negative	-	Negative	-
Accuracy-4	Negative	-	Negative	-
Accuracy-5	Negative	-	Negative	-
Accuracy-6	Negative	-	Negative	-
Accuracy-7	Positive	0.4691	Positive	0.1
Accuracy-8	Negative	-	Negative	-
Accuracy-9	Negative	-	Negative	-
Accuracy-10	Negative	-	Negative	-
Accuracy-11	Negative	-	Negative	-
Accuracy-12	Negative	-	Negative	-
Accuracy-13	Positive	1.8680	Negative	-

Accuracy-14	Negative	-	Negative	-
Accuracy-15	Negative	-	Negative	-
Accuracy-16	Negative	-	Negative	-
Accuracy-17	Positive	0.2415	Positive	0.23
Accuracy-18	Negative	-	Negative	-
Accuracy-19	Positive	0.0157	Negative	-
Accuracy-20	Negative	-	Negative	-
Accuracy-21	Negative	-	Negative	-
Accuracy-22	Negative	-	Negative	-
Accuracy-23	Negative	-	Negative	-
Accuracy-24	Negative	-	Negative	-
Accuracy-25	Positive	0.0241	Negative	-
Accuracy-26	Negative	-	Negative	-
Accuracy-27	Positive	0.1589	Positive	1.5
Accuracy-28	Negative	-	Negative	-
Accuracy-29	Negative	-	Negative	-
Accuracy-30	Negative	-	Negative	-
Accuracy-31	Positive	1.5837	Negative	1.8
Accuracy-32	Positive	1.6599	Negative	1.7

Table S5. Analytic sensitivity/limit of detection – serial dilution data. The first serial dilution targeted a blast percentage of 1%, followed by three 1:10 dilutions after that.

Sample	Target dilution %MRD	Measured %MRD
LOD-1	Native	77.4619
	1.000	0.9373
	0.1000	0.0876
	0.0100	0.0058
	0.0010	0.0000
LOD-2	Native	92.3015
	1.000	0.7352
	0.1000	0.0654
	0.0100	0.0044
	0.0010	0.0000
LOD-3	Native	30.6916
	1.000	0.8820
	0.1000	0.0986
	0.0100	0.0054
	0.0010	0.0000
